# A Conditional Mouse Mutant in the Tumor Suppressor *SdhD* Gene Unveils a Link between p21^WAF1/Cip1^ Induction and Mitochondrial Dysfunction

**DOI:** 10.1371/journal.pone.0085528

**Published:** 2014-01-20

**Authors:** África Millán-Uclés, Blanca Díaz-Castro, Paula García-Flores, Alicia Báez, José Antonio Pérez-Simón, José López-Barneo, José I. Piruat

**Affiliations:** 1 Instituto de Biomedicina de Sevilla, Hospital Universitario Virgen del Rocío/CSIC/Universidad de Sevilla. Seville, Spain; 2 Departamento de Hematología. Hospital Universitario Virgen del Rocío. Seville, Spain; 3 Centro de Investigación Biomédica en Red sobre Enfermedades Neurodegenerativas (CIBERNED), Madrid, Spain; Duke University Medical Center, United States of America

## Abstract

Mutations in mitochondrial complex II (MCII; succinate dehydrogenase, Sdh) genes cause familiar pheochromocytoma/paraganglioma tumors. Several mechanisms have been proposed to account for Sdh-mutation-induced tumorigenesis, the most accepted of which is based on the constitutive expression of the hypoxia-inducible factor 1α (Hif1α) at normal oxygen tension, a theory referred to as “pseudo-hypoxic drive”. Other molecular processes, such as oxidative stress, apoptosis, or chromatin remodeling have been also proposed to play a causative role. Nevertheless, the actual contribution of each of these mechanisms has not been definitively established. Moreover, the biological factors that determine the tissue-specificity of these tumors have not been identified. In this work, we made use of the inducible SDHD-ESR mouse, a conditional mutant in the *SdhD* gene, which encodes the small subunit of MCII, and that acts as a tumor suppressor gene in humans. The analysis of the Hif1α pathway in SDHD-ESR tissues and in two newly derived cell lines after complete *SdhD* loss -a requirement for hereditary paraganglioma type-1 tumor formation in humans- partially recapitulated the “pseudo-hypoxic” response and rendered inconsistent results. Therefore, we performed microarray analysis of adrenal medulla and kidney in order to identify other early gene expression changes elicited by *SdhD* deletion. Our results revealed that each mutant tissue displayed different variations in their gene expression profiles affecting to different biological processes. However, we found that the *Cdkn1a* gene was up-regulated in both tissues. This gene encodes the cyclin-dependent kinase inhibitor p21^WAF1/Cip1^, a factor implicated in cell cycle, senescence, and cancer. The two SDHD-ESR cell lines also showed accumulation of this protein. This new and unprecedented evidence for a link between *SdhD* dysfunction and p21^WAF1/Cip1^ will open new avenues for the study of the mechanisms that cause tumors in Sdh mutants. Finally, we discuss the actual role of Hif1α in tumorigenesis.

## Introduction

Germ-line mutations in the mitochondrial succinate dehydrogenase (Sdh) enzyme -also referred to as mitochondrial complex II (MCII)- or in its accessory units cause familial hereditary pheochromocytoma and paraganglioma [1], [2]. These are highly vascularized, mostly benign tumors that occur mainly in the adrenal gland and the carotid body. The MCII is composed of four nuclear-encoded subunits (Sdh-A, B, C and D) that couple the oxidation of succinate to fumarate in the Krebs cycle to the mitochondrial electron transport chain. The first gene found to be responsible for these types of tumors was *SDHD* [3]. Indeed, this gene was the first tumor suppressor gene identified as encoding a mitochondrial protein. Mutations on subunits B, C, A, as well as on the accessory protein SDHAF2, have subsequently also been implicated in paraganglioma, pheochromocytoma, renal cell carcinomas, and gastrointestinal tumors [2], [4], [5].

Several mechanisms have been proposed to account for the tumorigenic role of MCII mutations. One of the most accepted involves constitutive stabilization of the hypoxia inducible factor Hif1α. Due to its capacity to increase oxygen availability in tissues, as well as to activate anaerobic metabolism, Hif1α plays a fundamental role in tumor growth [6]–[10]. In Sdh-deficient cultured cells [11], [12] and tumors [13], succinate has been observed to accumulate in the cytosol. Succinate inhibits oxygen-dependent prolyl-hydroxylases (PHDs), responsible for targeting Hif1α to the von Hippel-Lindau (pVHL) ubiquitin-ligase for proteasome degradation in the presence of normal oxygen tension [14]. Hence, inhibition of PHDs could prevent Hif1α degradation in normoxic conditions, a situation termed to as “pseudo-hypoxia”, which would favor tumor formation and progression [9], [12], [13]. A role for oxidative stress in mitochondria-linked tumorigenesis has also been proposed. Thus, impairment of electron transfer at MCII would lead to electron leakage and/or possibly to a biased accumulation of the semi-reduced form of ubiquinone, which ultimately may contribute to mitochondrial reactive oxygen species (ROS) generation [15], [16]. Diffusion of ROS throughout the cell would eventually cause nuclear DNA damage and higher transforming mutation rates [15], [17], [18]. Additionally, free radicals generated under these conditions could also contribute to the stabilization of HIF1α by keeping the PHD cofactors, iron and α-ketoglutarate, in reduced form [14], [19], [20]. Another possibility is that accumulated succinate might inhibit other components of the α-ketoglutarate-dependent dioxygenase family such as histone demethylases, which might thereafter alter the expression of oncogenes and tumor suppressor genes [21]. Finally, inhibition of the normal pro-apoptotic activity of PHD-3 by succinate during development has been suggested to contribute to the pathogenesis of pheochromocytoma [22], [23].

Despite these lines of evidence, mostly obtained from cell culture studies, the precise molecular effects of MCII dysfunction *in vivo* remain essentially unknown. This is largely due to the lack of animal models that recapitulate defective Sdh-induced tumorigenesis. Homozygous knock-out mice for *SdhB* and *SdhD* are lethal at embryonic stages, and the heterozygotes do not present tumors or any other obvious pathology [24]–[26]. Conditional and tissue-specific *SdhD* mutant strains generated by our group also failed to show an increased predisposition to tumor occurrence [27]. These data suggest that the mechanisms of tumor transformation could differ between humans and rodents. In patients, tumor formation in heterozygous, paternally inherited *SDHD*-mutation carriers requires the loss of the maternal allele [3] in a phenomenon known as *loss of heterozygosity*. This parent-of-origin effect suggests a mechanism of genomic imprinting in the *SDHD* locus and/or other regions of the same chromosome [28]. Loss of the entire chromosome containing the gene has been observed in paraganglioma [29], which suggests that a "multiple-hit" process implicating other loci in the same chromosome may be required for tumor formation [30]. Given that chromosomal synteny is not conserved between the two species, different chromosomal arrangement could therefore account for the differences in tumor appearance between *SdhD*-mutant humans and mice.

In the present study, we further characterize the SDHD-ESR tamoxifen-inducible mouse model [27]. Based on the notion that the aforementioned proposed molecular mechanisms of tumorigenesis are triggered primarily by the complete loss of the *SdhD* gene, we consider this mouse an ideal model in which to study the early responses to the “second-hit” in paraganglioma, i.e., the loss of the remaining *SdhD* functional allele. For this purpose, we first analyzed the HIF1α pathway in SDHD-ESR mouse tissues as well as in newly derived cell lines. Additionally, and given that none of the hypothesis has been definitively established, we performed large-scale gene expression analysis in SDHD-ESR adrenal medulla and kidney tissue soon after *SdhD* deletion. Among other changes, we found that there is a differential response between these tissues, which might underlie the tissue-specificity of these tumors. However, we consistently observed that the p21^WAF1/Cip1^ encoding gene is up-regulated in both organs. This protein is implicated in many biological processes related to the cell cycle, survival, and cancer. The same up-regulation was observed in the cell lines. In light of the results obtained, we hypothesized that a check-point mechanism is activated upon total *SdhD* loss, which must be overcome by a subsequent third hit in order for the tumor transformation to occur. We also discuss the actual role of the Hif1α pathway in this process.

## Materials and Methods

### Mouse Strain, Husbandry and Treatment

The SDHD-ESR, with a *SdhD^flox/−^* Cre-ER™ genotype, tamoxifen-inducible mouse strain was generated as reported previously [27]. Littermates with *SdhD^flox/+^* and *SdhD^flox/−^* genotypes lacking CRE recombinase are referred to as wild-type homozygous (+/+) and heterozygous (+/−) mice, respectively, in this work. When indicated, results from both genotypes were pooled and assigned to a control group as no differences between them was found for the phenotypes tested. Routine genotyping was performed for the *SdhD* alleles by PCR with the following primers: 5′ AATTGTGCAGAAGTGAG-3′, 5′-GCTGCATACGCTTGATC-3′, 5′-CATCAAGGCTCACAGTC-3′. Mice were housed under temperature-controlled conditions (22°C) in a 12 h light/dark cycle, and provided with food and water *ad libitum*. Either high (100 µg/g for four days) or low (50 µg/g for two days) doses of tamoxifen dissolved in corn oil were administered by daily i.p. injections to 9–10 week-old animals.

### Ethics Statement

All experiments were performed in accordance with institutional guidelines approved by the ethics committee of the Virgen del Rocio University Hospital. The protocol was approved by the same committee according to the minute n° 02/2009.

### DNA Analysis

Genomic DNA was extracted from nuclear fractions resulting from mitochondrial preparations (see below) by overnight incubation at 37° in 0.1 M Tris-HCl (pH 8.5), 5 mM EDTA, 0.2% SDS, 0.2 M NaCl, 100 µg/ml proteinase K. The relative amount of the *SdhD^flox^* allele was estimated by quantitative PCR with the following primers: 5′-CTATGTAGGAGTCTGCAGCCAAGCT-3′, 5′-ACTCAAGGTCAGCCTCACCTACCTAT-3′, and normalized to the PCR product of the *GusB* gene.

### Mitochondrial Isolation and Enzymatic Complex Activities

Isolation of mitochondria from mouse kidney was performed as reported [25]. Mitochondrial complex I (MCI) and II activities were determined according to ref. 25 with slight modifications. Briefly, 30–50 µg of protein were assayed at 30°C. Samples were diluted 1∶4 in the assay reaction buffer (25 mM KH_2_PO_4_ pH 7.2, 5 mM MgCl_2_, 3 mM potassium cyanide, 2.5 mg/ml bovine serum albumin) and liquid nitrogen frozen-thawed three times before the assay. Rotenone-sensitive NADH-dehydrogenase activity was measured as the decrease in absorbance at 340 nm, referenced to 425 nm, due to oxidation of 130 µM NADH (Roche) in the presence of 3.6 µM antimycin and 130 µM ubiquinone-1 (Sigma). The absorbance was measured for 2 min before and after the addition of 5 µM rotenone (Sigma) to the reaction mixture. Differences between rates were considered when determining activity due to MCI. The succinate-ubiquinone-oxidoreductase activity of MCII was measured for a period of two minutes as the decrease in the absorbance at 600 nm due to the reduction of 50 µM 2,6-dichlorophenolindophenol (DCPIP) coupled to the reduction of 130 µM ubiquinone-1. The reaction was carried out in the presence of 3.6 µM antimycin, 5 µM rotenone and 10 mM succinate.

### Cell Lines

Cells were cultured in a humidified atmosphere of 5% CO_2_ at 37° in DMEM (Biowhittaker, BE12-614F) supplemented with 10% fetal bovine serum, MEM non-essential amino acids, 100 U/ml penicillin, 1 µg/ml streptomycin and 0.29 mg/ml L-glutamine. Mouse embryonic fibroblasts (MEFs) were obtained from E13.5-E15.5 embryos according to standard procedures. MEFs were immortalized by electroporation with the plasmid pEF321-T containing the SV40 large T antigen encoding gene (TAg), and seeding of the cells at clonal density. Stably transformed clones were subcultured, checked for the presence of TAg, and tested for continuous growth ability. Baby mouse kidney (BMK) cells were obtained from five-days-old mouse litters as reported [31]. For immortalization, BMK cells were electroporated with the plasmids pCMVE1A containing the viral oncogene E1A, and p53DD containing a dominant negative mutant allele of p53 (kindly gifted by Dr. Eileen White) according to a previously reported protocol [32]. Four-hydroxy-tamoxifen (Sigma) was added to the medium at 66 nM from a concentrated 2 mM stock in ethanol.

### RNA Analysis

Tissues were dissected and stored frozen at −80° until processing. Total RNA was prepared from mouse tissues and cultured cells with TRIzol® reagent (Life Technologies), according to the manufacturer’s directions for each type of sample. Total RNA from the adrenal medulla, surgically separated from cortex, was prepared using the RNeasy® microkit (Qiagen). Reverse transcription of mRNA was performed with the Superscript II reverse transcriptase kit (Life Technologies), and specific mRNA molecules were amplified by quantitative PCR in the presence of SYBR green® (Life Technologies) with the following primers for each gene: *SdhD*, 5′-CCAGCACATTCACCTGTCA-3′ and 5′-ATCAGCCCCAAGAGCAGAA-3′; *Vegf*, 5′-CGCAAGAAATCCCGGTTTAA-3′ and 5′-CAAATGCTTTCTCCGCTCTGA-3′; *Glut1*, 5′-CCAGCTGGGAATCGTCGTT-3′ and 5′-CAAGTCTGCATTGCCCATGAT-3′; *Phd3*, 5′-CAGACCGCAGGAATCCACAT-3′ and 5′-CATCGAAGTACCAGACAGTCATAGC-3′; *Cdkn1a*, 5′-TCCACAGCGATATCCAGACATT-3′ and 5′-CGGACATCACCAGGATTGG-3′. The *Arbp* housekeeping gene was used for normalization with the primers 5′-TCCAGGCTTTGGGCATCA-3′ and 5′-CTTTATCAGCTGCACATCACTCAGA-3′.

### High-throughput Gene Expression Studies

Total RNA was prepared from the adrenal medulla and kidney as above. Forty-five micrograms of RNA prepared from each kidney sample were further purified with the RNeasy® microkit (Qiagen). For microarray analysis, 200 ng (kidney) or 45 ng (adrenal medulla) of RNA were reverse-transcribed into cRNA and labeled with the Two-Color Microarray Low Input Quick Amp Labeling Kit (Agilent Technologies). Samples from 8 homozygous (+/+) individuals were pooled, labeled with cyanine 3-CTP (Cy3), and used as reference samples. Samples from 8 heterozygous (+/−) and 8 SDHD-ESR individuals were labeled with cyanine 5-CTP (Cy5) and used as test samples. All the individuals used were males. Labeled cRNA was mixed and hybridized against the oligonucleotide microarray slides Mouse GE 4×44 k V2 (Agilent Technologies). The microarrays were scanned in a GenePix® reader, with data acquired at wavelengths of 635 nm and 532 nm for Cy3 and Cy5, respectively. Acquired data were analyzed with the open-source Multi-Experiment Viewer software [33]. Values are represented as the log Cy5/Cy3 ratio of fluorescence intensities. the data generated in this study have been deposited in NCBI's Gene Expression Omnibus and are accessible through GEO Series accession number GSE52197 (http://www.ncbi.nlm.nih.gov/geo/query/acc.cgi?acc=GSE52197). The functional analysis was generated through the use of Ingenuity Pathways Analysis (IPA; Ingenuity Systems®, www.ingenuity.com).

### Western Blot

For protein preparation, tissues and cells were homogenized in ice-cold HEN buffer (5 mM EDTA, 250 mM NaCl, 50 mM Hepes pH 7.3, 5 mM DTT) containing 1 mM Na_3_VO_4_, 0.2% IGEPAL CA-630 (Sigma) and 1% protease inhibitor cocktail (Sigma). Homogenized samples were centrifuged for 30 min. at high speed in a microcentrifuge, after which protein containing supernatant was collected. Protein concentration was determined using a protein assay kit from Bio-Rad. From each sample, 50 µg of protein was separated by electrophoresis on SDS–polyacrylamide gels and electroblotted onto PVDF membranes. Blots were incubated in blocking solution (5% non-fat milk in PBS, 0.1% Tween-20 [PBS-T]), followed by overnight incubation with the following antibodies: anti-HIF1α (Cayman, CAY-1006421; dilution 1∶500); anti-Glut1 (Millipore, 07-1401; dilution 1∶800); anti-p21^WAF1/Cip1^ (Santa Cruz Biotechnology Inc., sc397; dilution 1∶200); and anti-β-actin (Abcam, ab6276; dilution 1∶5000). The membranes were then washed with PBS-T and incubated with either a HRP conjugated goat anti-rabbit IgG antibody (Thermo, 31460; dilution 1∶10000) or HRP conjugated sheep anti-mouse IgG antibody (ECL, NA931V; dilution 1∶10000). Antibody detection was performed with an enhanced chemiluminescence reaction (Clarity western ECL substrate; Bio-Rad).

### Statistical Analysis

Data are presented as mean ± standard error (SEM). Statistical significance was assessed by ANOVA with appropriate post-hoc analysis. For paired groups, either a Student’s t-test with a Levene test for homogeneity of variances in the case of normal distribution, or the nonparametric U-Mann Whitney test in the case of non-normal distributions, was applied. PASW18 software was used for statistical analysis. Statistical analysis of the microarray data was performed with the Multi-Experiment Viewer software [33] and the R programming language v3.2 (Vienna, Austria). False discovery rate (FDR) algorithm was used to identify genes in the datasets corresponding to +/− and SDHD-ESR groups differentially expressed respect to the +/+ genotype. Functional analysis with IPA was performed according to the tools provided in the software used.

## Results

### Validation and Dose-response Characterization of the SDHD-ESR Mouse

**Figure 1:**
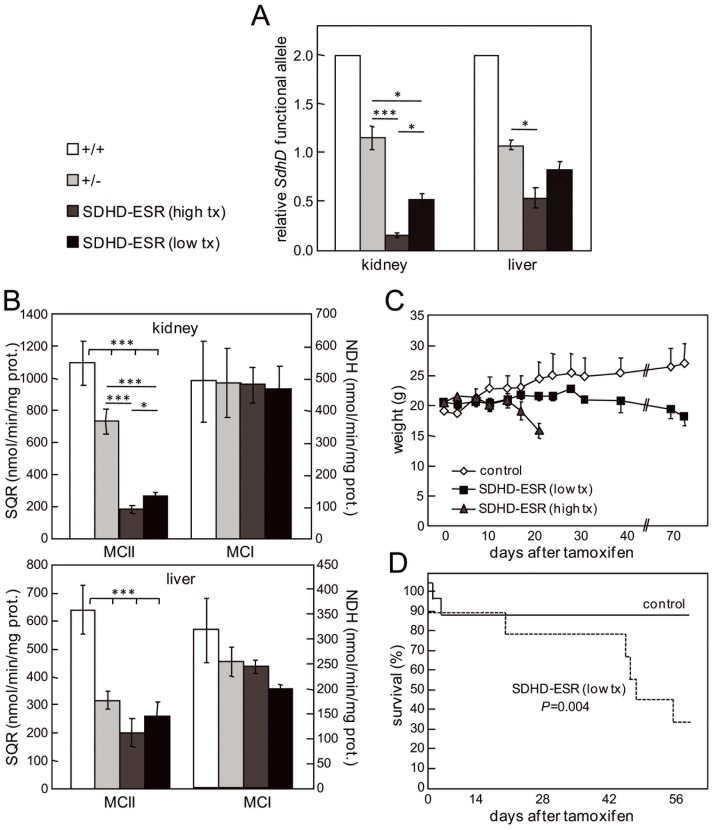
Validation and tamoxifen dose-response characterization of the inducible SDHD-ESR mouse. A. Relative amount of *SdhD* functional alleles (*SdhD* ^+^ or *SdhD^flox^*) in kidney and liver as determined by quantitative PCR of genomic DNA prepared 3 weeks after the start of injections of 100 µg/g, 4 times (high tx); or 50 µg/g, 2 times (low tx) tamoxifen. B. Succinate-ubiquinone oxidoreductase activity (SQR) of mitochondrial complex II (MCII) and NADH-dehydrogenase (NDH) activity of mitochondrial complex I (MCI) in kidney and liver 3 weeks after the first tamoxifen injection. C. Growth curves of 7-week-old mice after treatment with the same doses of tamoxifen. D. Survival curves of animals treated with the low dose of tamoxifen. Control group includes homozygous (+/+) and heterozygous (+/−) individuals without CRE recombinase, as no differences in the tested phenotypes were found between the two genotypes. Between 3 and 8 individuals per group were analyzed in each experiment. *, *P*≤0.05; **, *P*≤0.01; ***, *P*≤0.001.

The tamoxifen-inducible SDHD-ESR mutant mouse was generated as previously reported [27]. In order to assess the general CRE-mediated deletion of the *SdhD* gene, we analyzed the relative presence of the *SdhD^flox^* allele by quantitative PCR of total genomic DNA prepared from kidney and liver three weeks after the first tamoxifen injection (Figure 1A). The relative amount of functional *SdhD^flox^* allele in SDHD-ESR tissues decreased to about 16% and 54% in kidney and liver, respectively, with respect to the heterozygous *SdhD^flox/−^* without CRE (+/− genotype). When a lower dose of tamoxifen was administered, a lesser decrease was observed (Figure 1A). The stronger effect of tamoxifen on kidney versus liver as well as the dose dependency, were corroborated in functional studies by determining the succinate-ubiquinone oxidoreductase (SQR) activity of the MCII in the same tissues [27] (Figure 1B). The SQR activity in SDHD-ESR kidney and liver decreased to about 32% and 64% of the heterozygous level, respectively, whereas the NADH-dehydrogenase activity in these same tissue extracts was little or not significantly affected. This tamoxifen dose-dependency was translated to the general phenotype of mutant SDHD-ESR individuals as these animals stopped gaining weight after administration of the drug, and with the higher dose, even lost weight after two weeks (Figure 1C). High-dose tamoxifen-treated mutants were sacrificed before showing evident signs of suffering. Low-dose tamoxifen-treated mutants, although surviving longer than the high-dose treated animals, still had a shorter average life-span (7 weeks) than their wild-type littermates (Figure 1D). Since the treatment with the low dose of tamoxifen casts doubt about the efficient deletion of *SdhD* in all the tissues, the rest of experiments shown here were performed with the high dose.

### The “Pseudo-hypoxic Drive” is Partially Recapitulated in SDHD-ESR Tissues

**Figure 2:**
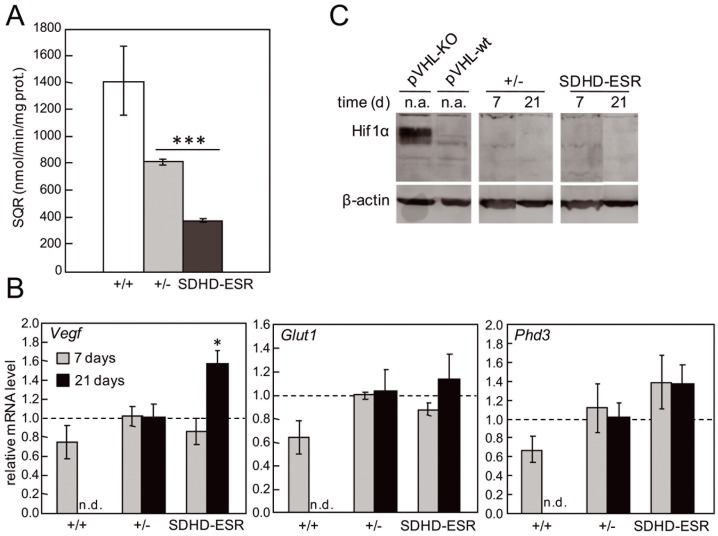
“Pseudo-hypoxic” response in SDHD-ESR mouse tissues. **A.** Succinate-ubiquinone oxidoreductase activity (SQR) in kidney 7 days after the start of the tamoxifen treatment. **B.** Relative mRNA level of *Vegf*, *Glut1*, and *Phd3* genes in kidney at 7 and 21 days after the start of the tamoxifen treatment. n.d.: non-determined. **C.** Western blot of Hif1α with protein extracts from kidney after tamoxifen treatment. Protein extracts from the pancreas of a β-cell-specific von Hippel-Lindau gene knock-out (VHL-KO) mouse and a wild-type littermate (VHL-wt) are loaded as controls. n.a.: non-applicable. Between 3 and 8 individuals per group were analyzed in each experiment. *, *P*≤0.05; ***, *P*≤0.001.

To address the possible activation of the “pseudo-hypoxic drive” mechanism [11]–[13], the expression of several HIF1α target genes was analyzed in wild-type (homozygous; +/+), heterozygous (+/−) and SDHD-ESR mutant tissues. We present here data obtained from kidney as this organ shows the most intense *SdhD* deletion of those analyzed. To prevent possible secondary effects due to the sustained lack of the gene in the animals three weeks after the start of the of treatment, we included in our analysis kidney samples obtained one week after the first tamoxifen injection. At this time point, SQR activity had already decreased considerably (Figure 2A). The messenger RNA levels of vascular endothelial growth factor (*Vegf*), glucose transporter 1 (*Glut1*), and prolylhydroxylase 3 (*Phd3*) genes were determined by RT-qPCR, revealing a non-statistically significant trend towards an increase in heterozygous animals compared with wild-type individuals (Figure 2B). However, when the remaining functional copy of *SdhD* was deleted, a significant induction of *Vegf* was observed in the SDHD-ESR kidney only three weeks after the start of injections, whereas for *Glut1* and *Phd3* their mRNA levels did not significantly increase further (Figure 2B). Moreover, no signs of Hif1α accumulation were observed, with this protein remaining undetected in mutant tissues as assessed by western blot at both time points (Figure 2C). As a control, protein extracts from a tissue-specific knock-out mouse for pVHL [34] showed induced expression of Hif1α. In addition, no increase was observed for any of the same tested genes in liver and brain (data not shown). Western blot of the same tissue extracts against the Hif2α antibody also produced no signal (data not shown). Together, these data suggest that activation of “pseudo-hypoxic drive” as a consequence of MCII depletion does not take place in a general and obvious manner in the analyzed mouse tissues.

#### The “pseudo-hypoxic drive” in SDHD-ESR-derived cell lines differs from that in tissues

**Figure 3:**
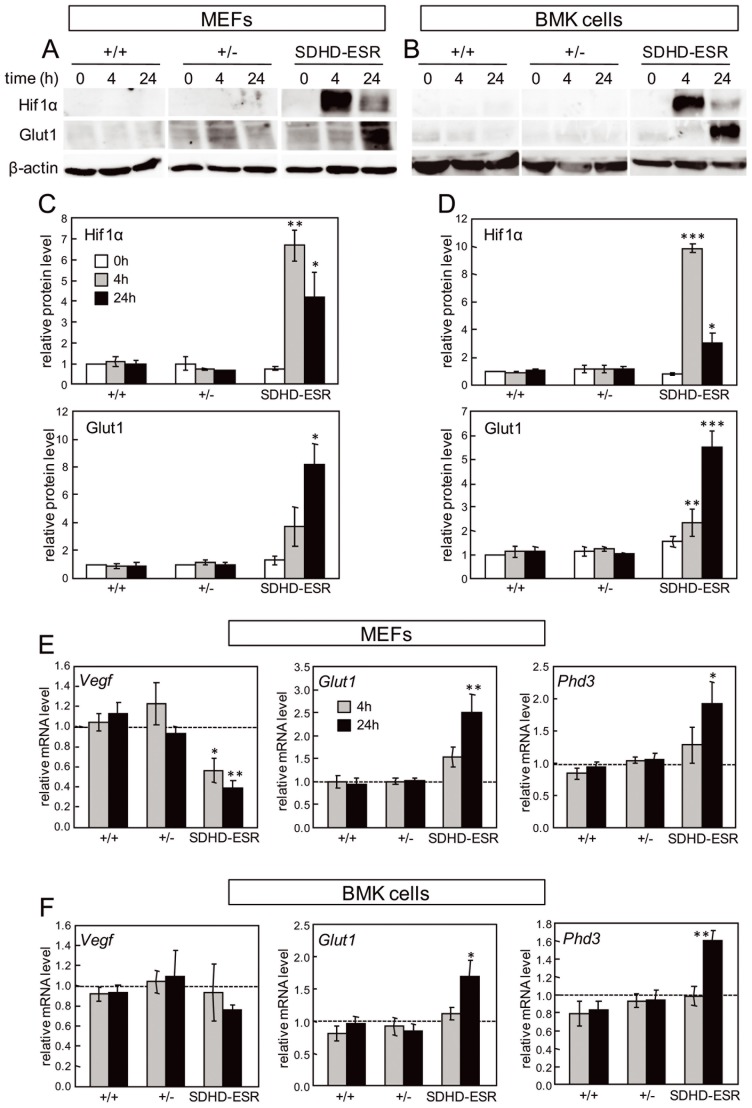
“Pseudo-hypoxic” response in SDHD-ESR MEFs and BMK cells. **A, B.** Western blot of Hif1α and Glut1 in total protein extracts of (**A**) mouse embryonic fibroblasts (MEFs) and (**B**) baby mouse kidney (BMK) cells obtained from SDHD-ESR mice and their homozygous *SdhD^flox/^* ^+^ (+/+) and heterozygous *SdhD^flox^* ^/−^ (+/−) littermates and cultured in medium supplemented with 4-hydroxy-tamoxifen for 4 or 24 hours. **C, D.** Quantification of relative Hif1α and Glut1 band intensities in (**C**) MEFs and (**D**) BMK cells normalized to β-actin signal. Results are the average ± SEM of three independent experiments. *, *P*≤0.05; **, *P*≤0.01; ***, *P*≤0.001; for 0 hours, i.e. in the absence of 4-hydroxy-tamoxifen, versus 4 or 24 hours of incubation in 4-hydroxy-tamoxifen. **E, F.** Messenger RNA levels of *Vegf*, *Glut1*, and *Phd3* genes in (**E**) MEFs and (**F**) BMK cells cultured in the same conditions. *, *P*≤0.05; **, *P*≤0.01; Two different immortalized clones were generated for each genotype and cell type giving the same results. Only results from one of each are shown.

As the diffusion kinetics of the drug throughout the mouse tissues could expand the *SdhD* deletion on time, thus hampering the detection of transitory Hif1α stabilization and the transcriptional activation of its target genes, we decided to establish cell lines from the SDHD-ESR mouse in which the accessibility of the cells to the drug is better controlled. We newly derived and immortalized the commonly used mouse embryonic fibroblasts (MEFs) cell type, as well as the epithelium-derived baby mouse kidney (BMK) cells, which are more suitable for the study of the biology of epithelial-derived tumors [31]. The BMK cells have recently proved to be useful for identifying synthetic lethal genes with the Krebs cycle enzyme fumarate hydratase [35], which is closely related to Sdh. Loss of *SdhD* was confirmed following addition of tamoxifen to the culture medium in two independent immortalized clones per cell type (data not shown). We tested the pseudo-hypoxia pathway in our immortalized MEF and BMK cell lines after tamoxifen exposure. Whereas in heterozygous lines no differences were found in either Hif1α or Glut1 protein levels with respect to the wild type, the SDHD-ESR mutant line underwent accumulation of both proteins, although with different kinetics of induction (Figure 3A–D). When mRNA levels were determined for some Hif1α-target genes, *Phd3* and *Glut1* expression were found to be induced in the SDHD-ESR cell line, whereas *Vegf* expression was not, or was even significantly down-regulated (Figure 3E, F). These results, although indicative of a “pseudo-hypoxic drive” effect on *SdhD*-null cell lines, contrast strongly with the observations made in mouse tissues.

#### Microarray studies in two mouse tissues reveal differential responses to SdhD deletion

**Figure 4:**
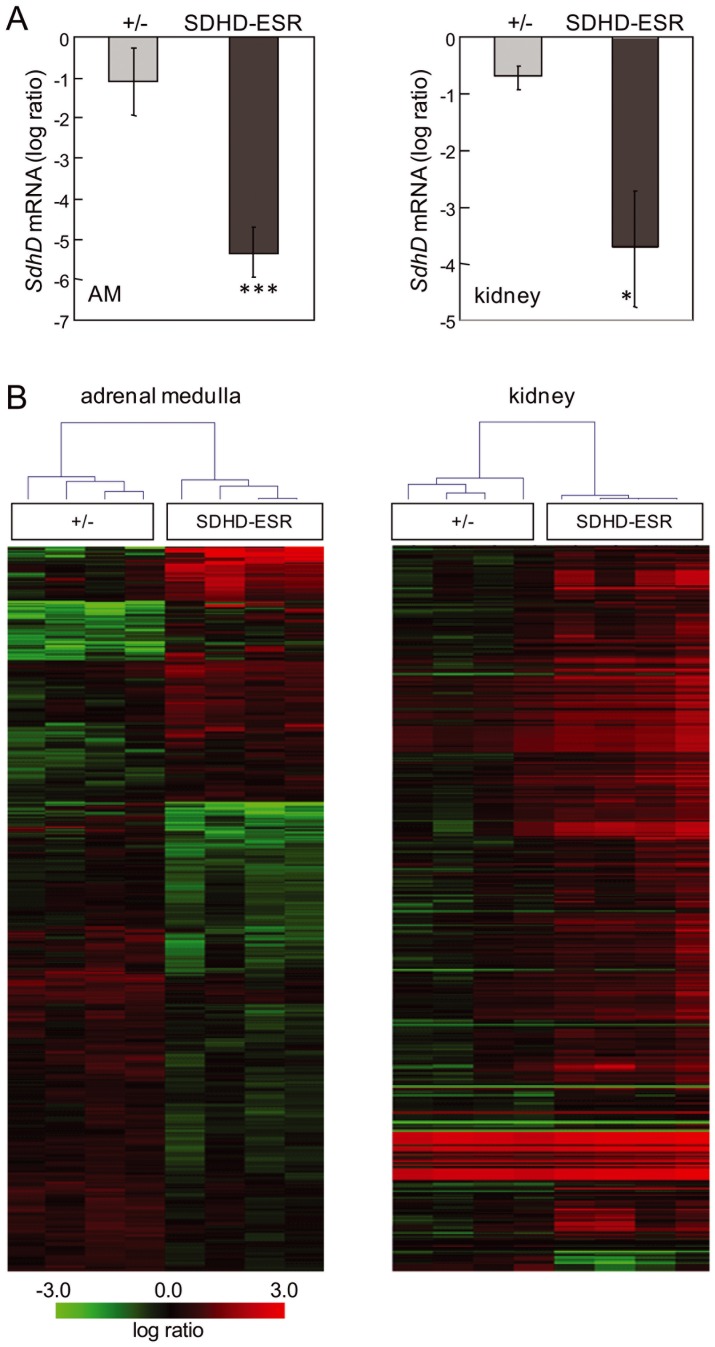
Microarray analysis. **A.** *SdhD* mRNA levels in heterozygous (+/−) and SDHD-ESR adrenal medulla and kidney relative to wild-type (+/+) tissues 7 days after the start of the tamoxifen treatment, as obtained from the corresponding microarray feature (Gene Bank accession n° NM_025848). *, *P*≤0.05; ***, *P*≤0.001. The number of samples is 8 per group. **B.** Supervised hierarchical clustering of adrenal medulla (AM) and kidney samples based on genes that showed significant differences in their expression level. The heatmap and the hierarchical tree are shown for 8 samples, grouped in pairs, per genotype.

**Figure 5:**
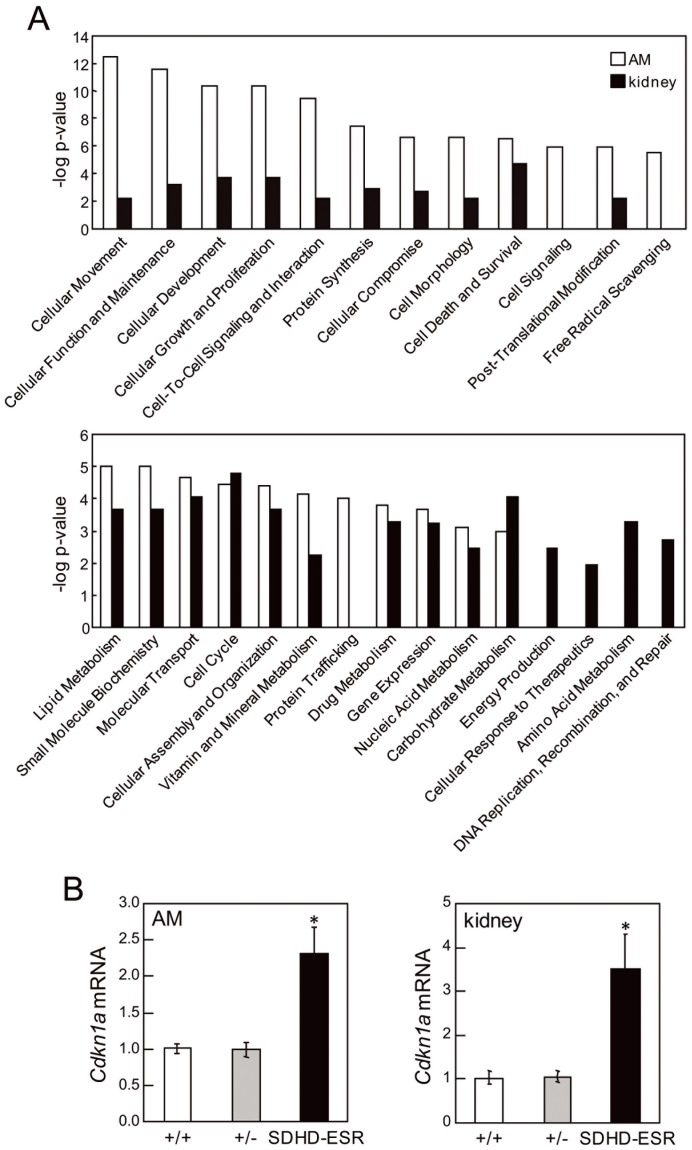
Functional analysis and validation of the microarray. **A.** Comparative functional analysis of datasets generated from adrenal medulla (AM) and kidney samples from SDHD-ESR mice. The significance of each molecular and cellular function is indicated by –log of the p-value. **B.** Quantitative RT-PCR of RNA samples used in the microarray study based on specific primers for amplification of the *Cdkn1a* mRNA.

With the aim of identifying alternative molecular pathways altered upon SdhD deletion, and to determine if some tissue-specific features could condition the transcriptional response to MCII depletion, we performed high-throughput gene expression analysis of kidney and adrenal medulla tissue. The adrenal medulla was chosen because it is one of the main pheochromocytoma/paraganglioma target tissues. The kidney was chosen not only because of the more intense effect on SdhD deletion exerted by tamoxifen, but also due to the finding that renal cell carcinoma, although much less frequently, is also associated with Sdh-mutations. To minimize secondary effects on gene expression, we analyzed samples obtained one week after the first tamoxifen injection. At this time point, SdhD mRNA levels in both tissues had already decreased considerably [27] (Figure 4A). It is worth to mention that at this time-point no major histological abnormalities were detected for any of the tissues analyzed. For large-scale gene expression analysis we performed two-color microarray hybridization. Total RNA from wild-type individuals was labeled with the fluorescent dye Cy3 and used as the reference sample. Total RNA from either heterozygous (+/−) or SDHD-ESR individuals was labeled with the fluorescent dye Cy5 and used as the test samples. In this way, the relative abundance of each transcript was monitored by the ratio between the two fluorescence intensities in their corresponding spot-features. Among the genes that were significantly affected in tissues from SDHD-ESR animals (FDR≤0.05) those showing statistically significant differences in expression between +/− and SDHD-ESR groups (P≤0.05) were identified and analyzed. Remarkably, some genes that have previously been shown to respond to hypoxia in a HIF1α-dependent manner were not overexpressed in null SdhD deficient tissues (table 1). Supervised hierarchical clustering of samples based on the significant genes demonstrated different gene expression profile changes between the adrenal medulla and kidney (Figure 4B). For functional analysis, the entire datasets containing gene identifiers and their corresponding expression values were uploaded into the Ingenuity Pathway Analysis (IPA) application. A log ratio ±0.2 cut-off was set to identify genes whose expression was differentially regulated. As an internal control we detected the aforementioned decreases to about half of the SdhD mRNA level in the heterozygous (+/−) tissues, and to 2% and 7% in the adrenal medulla and kidney, respectively, in the SDHD-ESR mice (Figure 4A; table 2). As our initial objective was to identify biological pathways exclusively affected by complete loss of SdhD, functional analysis of their respective datasets was performed focusing on SDHD-ESR samples. Most of the significant high-level molecular and cellular functions found to be affected differed greatly between the adrenal medulla and kidney (Figure 5A). The highest scores were given to the adrenal medulla dataset and were categorized in cellular movement, maintenance, development, growth and proliferation categories, as well as cell-to-cell signaling and interaction, among others. On the other hand, functions found to be solely affected in the kidney were energy production, cellular response to therapeutics, amino acid metabolism, and DNA replication, recombination and repair. When these functional categories were analyzed using an algorithm that predicts the effect of gene expression changes on specific functions, the significant results in adrenal medulla samples (z-score ≥2, or ≤ −2) predicted decreases in white blood cell movement, viability, proliferation and production of antibody (table 3), which is consistent with an inhibition of the inflammatory response. In contrast, specific functions significantly predicted to increase in the kidney were cell movement, survival, differentiation, and death, as well as carbohydrate, lipid and amino acid metabolism (table 4). Despite these differences, some few genes were found to be affected in both tissues. They are ACOT1, Camk2b, CDKN1A, ESR1, HSD11B1, LONRF3, MST1R, PRODH2, SHANK3, STAT3, SMARCD3, and TEX12. Among them, Cdkn1a was significantly up-regulated and showed a log ratio of intensities ≥1 in both the adrenal medulla and kidney of the SDHD-ESR mutant (table 2). This effect was validated by quantitative RT-PCR (Figure 5B). The Cdkn1a gene encodes the cyclin-dependent kinase inhibitor p21^WAF1/Cip1^, and is well known to be implicated in many processes such as the cell cycle, cell proliferation, senescence and cancer [36], [37].

**Table 1:** Expression levels of HIF1α-mediated hypoxia responsive genes.

	Adrenal medulla		Kidney	
	+/−	SDHD-ESR	+/−	SDHD-ESR
*Glut1*	−0.099±0.216	−0.323±0.304	−0.176±0.207	−0.455±0.276
*HK2*	0.722±0.565	−0.262±0.449	−0.061±0.201	0.021±0.323
*LDHA*	−0.042±0.517	−0.041±0.413	1.959±0.813	1.481±0.841
*PDK1*	−0.315±0.237	0.126±0.127	−0.043±0.153	−0.785±0.153
*Vegf*	−0.076±0.192	−0.133±0.203	−0.391±0.175	−0.621±0.216

Date are expressed as the log ratio ± SEM between either the homozygous (+/−) or the inducible SDHD-ESR mutant and the wild type (+/+) expression levels for each gene in each tissue as obtained from the microarray analysis. *Glut1*: glucosyltransferase 1 (NM_172380), *HK2*: Hexokinase 2 (NM_013820), *LDHA*: Lactate dehydrogenase A (NM_001136069), *PDK1*: Pyruvate dehydrogenase kinase 1 (NM_172665), *Vegf*: Vascular endothelial growth factor (NM_001025257).

**Table 2:** Most strongly up- and down-regulated genes in SDHD-ESR tissues.

Adrenal medulla				
Log Ratio^a^	ID	Symbol	Entrez Gene Name	FDR
3.035	NM_025687	*TEX12*	testis expressed 12	0.043
2.541	NM_007956	*ESR1*	estrogen receptor 1	0.033
2.349	NM_001111099	*CDKN1A*	cyclin-dependent kinase inhibitor 1A (p21, Cip1)	0.006
2.018	NM_011356	*FRZB*	frizzled-related protein	0.014
1.701	NM_170599	*IGSF11*	immunoglobulin superfamily, member 11	0.005
1.629	NM_011228	*RAB33A*	RAB33A, member RAS oncogene family	0.002
1.595	NM_001001979	*MEGF10*	multiple EGF-like-domains 10	0.033
1.248	NM_023529	*MS4A10*	membrane-spanning 4-domains, subfamily A, member 10	0.006
1.062	NM_028733	*PACSIN3*	protein kinase C and casein kinase substrate in neurons 3	2.42E-7
1.040	NM_001081147	*OXTR*	oxytocin receptor	2.25E-4
−5.326	NM_025848	*SDHD*	succinate dehydrogenase complex, subunit D	2.52E-5
−2.129	NM_007988	*FASN*	fatty acid synthase	2.77E-4
−1.673	NM_001145164	*Tgtp1/Tgtp2*	T cell specific GTPase 1	4.78E-5
−1,505	NM_010174	*FABP3*	fatty acid binding protein 3	0.022
−1.458	NM_010501	*IFIT3*	interferon-induced protein with tetratricopeptide repeats 3	0.004
−1.311	NM_013653	*CCL5*	chemokine (C-C motif) ligand 5	1.75E-4
−1.186	NM_009930	*COL3A1*	collagen, type III, alpha 1	2.85E-5
−1.134	NM_018738	*Igtp*	interferon gamma induced GTPase	3.24E-5
−1.062	NM_013585	*PSMB9*	proteasome (prosome, macropain) subunit, beta type, 9	1.78E-4
−1.037	NM_007572	*C1QA*	complement component 1, q subcomponent, A chain	1.50E-7
**Kidney**				
**Log Ratio** a	**ID**	**Symbol**	**Entrez Gene Name**	**FDR**
2.931	NM_007956	*ESR1*	estrogen receptor 1	8.91E-4
2.623	NM_008239	*FOXQ1*	forkhead box Q1	6.26E-5
2.245	NM_080852	*Slc7a12*	solute carrier family 7, member 12	6.40E-4
2.163	NM_138595	*GLDC*	glycine dehydrogenase (decarboxylating)	4.88E-5
2.066	NM_025687	*TEX12*	testis expressed 12	2.03E-6
1.964	NM_007669	*CDKN1A*	cyclin-dependent kinase inhibitor 1A (p21, Cip1)	4.52E-4
1.915	NM_008256	*HMGCS2*	3-hydroxy-3-methylglutaryl-CoA synthase 2	2.69E-3
1.869	NM_010196	*FGA*	fibrinogen alpha chain	0.015
1.855	NM_145368	*Acnat1/Acnat2*	acyl-coenzyme A amino acid N-acyltransferase 1	0.002
1.774	NM_016668	*BHMT*	betaine-homocysteine S-methyltransferase	0.032
−3.737	NM_025848	*SDHD*	succinate dehydrogenase complex, subunit D	3.56E-6
−2.479	NM_011315	*Saa3*	serum amyloid A 3	6.23E-4
−2.380	AK143946	*ACSM3*	acyl-CoA synthetase medium-chain family member 3	7.98E-5
−1.546	NM_001081688	*TMPRSS9*	transmembrane protease, serine 9	5.77E-6
−1.306	NM_009127	*SCD*	stearoyl-CoA desaturase (delta-9-desaturase)	5.93E-5
−1.201	NM_146086	*PDE6A*	phosphodiesterase 6A, cGMP-specific, rod, alpha	4.14E-4
−1.007	NM_145360	*IDI1*	isopentenyl-diphosphate delta isomerase 1	1.12E-4
−0.829	NM_009892	*Chi3l3/Chi3l4*	chitinase 3-like 3	6.23E-4
−0.793	NM_181849	*FGB*	fibrinogen beta chain	0.015
−0.637	NR_002860	A130040M12Rik	RIKEN cDNA A130040M12 gene	1.76E-4

a ; Values >0 indicate up-regulated genes. Values <0 indicate down-regulated genes. FDR: False discovery rate.

**Table 3:** Biological functions predicted to be affected in the SDHD-ESR adrenal medulla.

Functions Annotation	p-Value	Predicted Activation State	Activatio z-score	# Genes	Genes
leukocyte migration	2,95E-10	Decreased	−2,618	41	*ADORA3, ANGPTL2, CASP1, CD1D, CD48, CDKN1A, CRP, CTSC, CX3CR1, CXCL14, CXCL9, CXCR3, CXCR5, CYSLTR1, DCN, FCER1G, FCGR2B, FUT4, HCK, HDC, HEBP1, IL1B, IL4R, LDLR, LGALS3, LILRB3, MDK, MMP9, MPP1, MYO1F, NCKAP1L, PRKCD, RAC2, SELPLG, SEMA4D, STAT3, SYK, TIRAP, TLR2, TYROBP, VIPR1.*
cell viability of leukocytes	7,94E-04	Decreased	−2,617	11	*EGF, FCER1G, IL1B, IL2RG, LGALS3, RAC2, SEMA4D, SHH, STAT3, SYK, TYROBP.*
degranulation of mast cells	7,86E-07	Decreased	−2,574	11	*ADORA3, DHCR7, FCER1G, FCGR2B, HCK, IL1B, IL4R, RAC2, SYK, TLR2, VAV1.*
degranulation of phagocytes	3,79E-08	Decreased	−2,572	13	*ADORA3, ANXA3, DHCR7, FCER1G, FCGR2B, HCK, IL1B, IL4R, MYO1F, RAC2, SYK, TLR2, VAV1.*
infiltration of cells (leukocytes)	1,09E-05	Decreased	−2,507	19	*CASP1, CD48, CXCR5, DCN, FCER1G, FCGR2B, FUT4, HCK, IL1B, IL4R, LDLR, LGALS3, MDK, MMP9, PRKCD, RAC2, SELPLG, STAT3, TLR2.*
cell movement of blood cells (macrophages, granulocytes, antigen presenting cells, leukocytes)	8,78E-11	Decreased	−2,477	42	*ADORA3, ANGPTL2, CASP1, CD1D, CD48, CDKN1A, CRP, CTSC, CX3CR1, CXCL14, CXCL9, CXCR3, CXCR5, CYSLTR1, DCN, FCER1G, FCGR2B, FUT4, GJA1, HCK, HDC, HEBP1, IL1B, IL4R, LDLR, LGALS3, LILRB3, MDK, MMP9, MPP1, MYO1F, NCKAP1L, PRKCD, RAC2, SELPLG, SEMA4D, STAT3, SYK, TIRAP, TLR2, TYROBP, VIPR1*
cell death of immune cells	1,33E-06	Decreased	−2,458	27	*ADORA3, C1QA, CASP1, CASP3, CD27, CD79B, CDKN1A, CRP, EGF, FCER1G, FCGR2B, FLT3, IL1B, IL2RG, LDLR, LGALS3, PRKCD, RAC2, SEMA4D, SHH, ST6GAL1, STAT3, SYK, TLR2, TNFAIP8L2, TYROBP, VAV1.*
proliferation of T lymphocytes	6,66E-06	Decreased	−2,360	26	*ARHGDIB, BATF, CASP3, CD1D, CD27, CD48, CD83, CDKN1A, CRP, EBI3, FCER1G, FCGR2B, HLA-DRB1, IL1B, IL2RG, IL4R, MMP9, NCKAP1L, RAC2, SHH, STAT3, SYK, TLR2, TRAF5, TYROBP, VAV1.*
activation of T lymphocytes	2,81E-03	Decreased	−2,337	12	*CD1D, CD48, CDKN1A, DCT, FCER1G, IL1B, IL2RG, LDLR, SEMA4D, STAT3, TLR2, VAV1.*
phagocytosis of cells	4,81E-04	Decreased	−2,263	11	*ANXA3, C1QA, CRP, FCER1G, FCGR2B, HCK, LGALS3, RAC2, SYK, TLR2, VAV1.*
adhesion of granulocytes	1,42E-03	Decreased	−2,213	6	*HCK, IL1B, LGALS3, MMP9, SELPLG, VAV1.*
production of superoxide	1,07E-04	Decreased	−2,208	8	*CXCL9, CYBA, GCH1, HCK, IL1B, RAC2, TLR2, TYROBP.*
immune response of antigenpresenting cells	1,60E-03	Decreased	−2,160	8	*CD1D, FCER1G, FCGR2B, HCK, LGALS3, MARCH1, SYK, TLR2.*
migration of cells	1,11E-06	Decreased	−2,157	53	*ABI3, ADORA3, ANGPTL2, ANXA3, CASP1, CD1D, CD48, CDKN1A, COL3A1, CRP, CSPG4, CTSC, CX3CR1, CXCL14, CXCL9, CXCR3, CXCR5, CYSLTR1, DCN, EGF, FCER1G, FCGR2B, FUT4, GJA1, HCK, HDC, HEBP1, IL1B, IL4R, LDLR, LGAL S3, LILRB3, MDK, MMP9, MPP1, MYO1F, NCKAP1L, PRKCD, RAC2, SELPLG, SEMA4D, SHH, SORT1, ST8SIA4, STAT3, SYK, TGFB3, THRB, TIRAP, TLR2, TMSB10/TMSB4X, TYROBP, VIPR1.*
quantity of IgM	1,83E-03	Decreased	−2,157	8	*BATF, CD83, CDKN1A, FCGR2B, IL2RG, LDLR, PRKCD, TRAF5.*
production of antibody	2,38E-08	Decreased	−2,077	22	*BATF, C1QA, CD1D, CD83, CDKN1A, CXCL9, FCER1G, FCGR2B, IL2RG, IL4R, LDLR, LGALS3, MMP9, PRKCD, SEMA4D, TIRAP, TLR1, TLR2, TNFAIP8L2, TRAF5, TYROBP, VAV1.*
phagocytosis of blood cells	1,41E-04	Decreased	−2,043	9	*CRP, FCER1G, FCGR2B, HCK, LGALS3, RAC2, SYK, TLR2, VAV1.*

**Table 4:** Biological functions predicted to be affected in the SDHD-ESR kidney.

Functions Annotation	p-Value	Predicted Activation State	Activation z-score	# Genes	Genes
cell movement (migration of cells)	4,29E-03	Increased	3,227	52	*ANGPTL3, B4GALT1, BMP4, BTC, C3, CALML3, CD8A, CDKN1A, CEBPD, Chi3l3/Chi3l4, EPHB3, ERF, ESR1, FGA, FGB, FOSL2, HP, ID1, ID3, IL15RA, IL33, IL6R, IRS2, Klra4 (includes others), LCN2, MAP3K5, MCAM, MST1R, MYC, NCOA4, NFKBIA, NQO1, OSMR, Pde4d, PPARA, PRAP1, PRNP, PTPN1, PTPRJ, REST, RGS3, SCNN1A, SLC1A3, SLC37A4, SOCS2, SOCS3, SPP1, STAT3, STIM1, TNFRSF21, TNFRSF9, XDH.*
cell viability and survival (apoptosis)	1,85E-04	Increased	3,020	42	*ABCC3, ATF2, BMP4, BNIP3, BTC, CD8A, CDKN1A, CEBPD, CISH, ESR1, FA2H, FGF18, FHIT, IL33, IL6R, IRS2, LCN2, MAP3K5, MCAM, MGST1, MMS22L, MYC, MYOD1, NFIL3, NFKBIA, OGG1, PPP2R2B, PRNP, PTPN1, SLC1A3, SLC22A8, SLC37A4, SOCS2, SOCS3, SPP1, STAT3, TNFRSF9, UNG, XDH, XPA.*
differentiation of cells (tubulation of endothelial cells)	4,92E-04	Increased	2,886	56	*ATF2, BGLAP, BMP4, BNIP3, BTC, BTG1, C3, CD8A, CDKN1A, CEBPD, CLCF1, CYTL1, EPHB3, ERF, ESR1, EYA1, FGF18, FLVCR1, FOSL2, HSD11B1, ID1, ID3, IFRD1, IHH, IKZF4, IL15RA, IL33, IL6R, IRS2, LCN2, MAFF, MAP3K5, MST1R, MYC, MYOD1, NFIL3, NFKBIA, PPARA, PRNP, PTPN1, PTPRJ, REST, RPS3A, SCD, SLC1A3, SLC37A4, SMAD6, SMARCD3, SOCS2, SOCS3, SPP1, STAT3, STIM1, TNFRSF9, TRIB3, XDH.*
interphase	3,13E-03	Increased	2,641	23	*ATF2, BMP4, BTG1, Camk2b, CDKN1A, CEBPD, ESR1, FHIT, ID1, ID3, IL6R, MAP3K5, MMS22L, MYC, NFKBIA, PPARA, PRNP, SIAH1, SMAD6, STAT3, STK38L, XPA, ZBTB10.*
transport of carbohydrate	5,55E-04	Increased	2,467	10	*ABCC3, B4GALT1, C3, IRS2, MGAT4A, PPP1R3B, SLC1A3, SLC37A4, SLC5A1, TRIB3.*
oxidation of lipid (beta-oxidation of fatty acid)	7,60E-05	Increased	2,203	12	*C3, CYP27A1, CYP4A11, HACL1, HSD11B1, IRS2, PNPLA2, PON1, PPARA, Rdh1 (includes others), SAT1, SCD.*
metabolism of amino acids	4,71E-03	Increased	2,183	8	*BHMT, DDC, GLDC, GLS, KYNU, MYC, PPARA, SLC1A3.*
proliferation of cells (generation of lymphocytes; cytostasis; proliferation of mammary tumor cells and neuroblasts)	5,99E-03	Increased	2,101	84	*ABCC3, APOD, Art2a-ps/Art2b, ATF2, B4GALT1, BMP4, BNIP3, BTC, BTG1, C3, CACNA1G, CBR1, CD8A, CDCA4, CDKN1A, CEBPD, CISH, CLCF1, CYP20A1, EPHA5, EPHB3, ERAL1, ERF, ESR1, EYA1, FA2H, FGA, FGF18, FHIT, FOSL2, GP2, HSD11B1, HTR3A, ID1, ID3, IHH, IL15RA, IL33, IL34, IL6R, IRS2, ITIH4, LCN2, MAFF, MAP3K5, MCAM, MST1R, MYC, MYOD1, NCOA4, NFKBIA, NMB, NQO1, OSMR, PDXK, PPARA, PRNP, PTPN1, PTPRJ, REST, RING1, RPS3A, SAT1, SF1, SIAH1, SLC1A3, SLC20A1, SMAD6, SMARCD3, SOCS2, SOCS3, SPP1, STAT3, STIM1, STK38L, SULF2, TNFRSF21, TNFRSF9, TRIM25, TSC22D1, UTP20, VPS53, XDH, XPA.*
necrosis	3,03E-04	Increased	2,003	69	*ALDH2, ALDH3B1, Art2a-ps/Art2b, ATF2, ATXN7, BMP4, BNIP3, BTC, BTG1, C3,CD8A, CDKN1A, CEBPD, CISH, CLCF1, EHMT1, ESR1, FAM134B, FAM176C, FGA, FGF18, FHIT, GP2, ID1, ID3, IFRD1, IL15RA, IL33, IL6R, IRS2, ITIH4, Klra4 (includes others), LCN2, MAP3K5, MMS22L, MST1R, MYC, MYOD1, NFIL3, NFKBIA, NQO1, OGFOD1, PNPLA2, PPARA, PPP2R2B, PRNP, PTPN1, REST, RGS3, RPS3A, SAT1, SCD, SEMA7A, SGCG, SIAH1, SLC1A3, SLC20A1, SMAD6, SOCS3, SPP1, STAT3, STIM1, TNFRSF21, TNFRSF9, TRIB3, TSC22D1, UNG, XDH, XPA.*
concentration of triacylglycerol	2,35E-08	Decreased	−2,520	21	*ANGPTL3, APOD, BGLAP, BHMT, C3, CIDEC, CYP27A1, HSD11B1, IFRD1, IRS2, MGAT4A, MYC, NQO1, PNPLA2, PON1, PPARA, PTPN1, SCD, SLC37A4, SPP1, XDH.*
binding of DNA	7,09E-07	Decreased	−2,635	28	*ATF2, BTAF1, CDKN1A, CEBPD, CISH, ERF, ESR1, FOSL2, HIF3A, HP, ID1, ID3, IL33, IL6R, MYC, MYOD1, NFKBIA, PPARA, PTPN1, REST, SCD, SOCS2, SOCS3, SPP1, STAT3, TRIB3, XPA, ZBTB10.*

#### p21WAF1/Cip1 expression is induced in SDHD-ESR-derived cell lines

**Figure 6:**
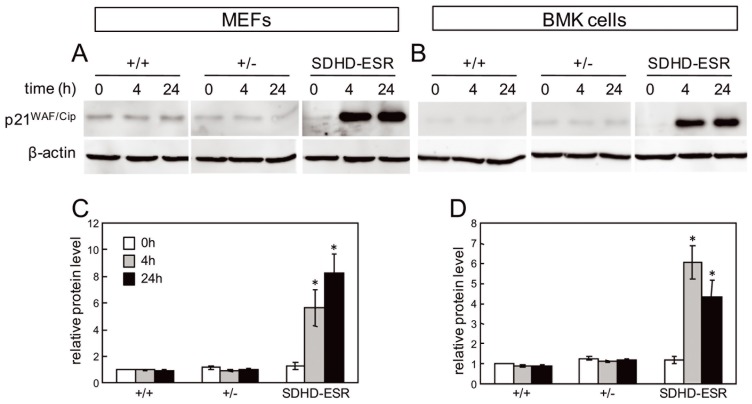
Analysis of p21^WAF/Cip^ in SDHD-ESR-derived cell lines. **A, B.** Western blot of p21^WAF1/Cip1^ in total protein extracts of (**A**) MEFs and (**B**) BMK cells obtained from SDHD-ESR mice and their homozygous *SdhD^flox^* ^/+^ (+/+) and heterozygous *SdhD^flox/−^* (+/−) littermates and cultured in medium supplemented with 4-hydroxy-tamoxifen for 4 or 24 hours. Quantification of relative p21^WAF1/Cip1^ band intensities in (**C**) MEFs and (**D**) BMK cells normalized to β-actin signal. Results are the average ± SEM of three independent experiments. Two different immortalized clones were generated for each genotype and cell type giving the same results. *, *P*≤0.05; ***, *P*≤0.001; for 0 hours, i.e. in the absence of 4-hydroxy tamoxifen, versus 4 or 24 hours of incubation in 4-hydroxytamoxifen.

To confirm the up-regulation of p21^WAF1/Cip1^ in *SdhD*-deficient cells, we analyzed its expression in the MEF and BMK cell lines described above. In both cell types, p21^WAF/Cip^ expression was strongly up-regulated in the mutant SDHD-ESR cell lines 4 hours after tamoxifen-induced *SdhD* deletion, and the amount of protein remained increased after 24 hours (Figure 6). In contrast, there were no signs of accumulated protein in the heterozygous cell lines at any time.

## Discussion

In this study, we aimed to identify the molecular events triggered after the second hit of the process of MCII mutation-induced tumorigenesis by inducing loss of the second *SdhD* allele *in vivo*. To achieve this, we made use of the conditional tamoxifen-inducible SDHD-ESR mouse mutant [27]. This model does not seem to recapitulate the proposed “pseudo-hypoxic” response to MC-II depletion [9], [12], [13] in a general and consistent manner. Indeed, two newly derived and immortalized SDHD-ESR cell lines showed a response to some extent incoherent with the results obtained in animal tissues. Therefore, we aimed at identifying other molecules potentially responding to the *SdhD* deletion. High-throughput analysis of gene expression allowed us to identify the *Cdkn1a* gene, which encodes the cyclin-dependent kinase inhibitor p21^WAF1/Cip1^, as being up-regulated in two *SdhD*-deficient tissues. We also evaluated whether or not p21^WAF1/Cip1^ was also affected in the SDHD-ESR derived MEFs and BMK cells. In both cell types we observed that, like in tissues, complete loss of *SdhD* led to accumulation of this protein whereas no increase was observed in heterozygous *SdhD* ^+/−^ cells. Our observations unveil a new link between the loss of the tumor suppressor gene *SdhD* and p21^WAF1/Cip1^ activation that occurs in a general manner.

Regarding the biological consequences of this observation, it is widely accepted, although not demonstrated, that after the loss of the two copies of the *SDHD* gene has occurred, a third hit seems to be required for the tumorigenic process to start [30]. This would be a requirement specific for *SDHD,* as tumor development in *SDHB*, *SDHC* and *SDHA* appear to follow a two-hit kinetics. To date, the nature of this hypothetical third hit is completely unknown. In the light of our results, and given the implication of p21^WAF1/Cip1^ in the cell cycle, proliferation, and senescence, we propose that the *SdhD*-mutation-induced p21^WAF1/Cip1^ up-regulation could represent a checkpoint mechanism activated upon MCII failure. Thus, any subsequent molecular event that causes the cell division machinery to by-pass this checkpoint would drive the cells to replicative catastrophe, accumulating mutations and eventually resulting in tumor transformation. Our findings, therefore, open new avenues for the identification of candidate genes involved in the cell cycle and growth, genome integrity, and other processes, whose mutations could result in the oncogenic transformation of Sdh-deficient tissues.

Previous reports in humans, based on gene expression profiling and unsupervised hierarchical clustering, demonstrate a tight association between pheochromocytomas with VHL and SDH mutations, which distinguishes them from pheochromocytomas with MEN2, RET, and NF1 mutations [10], [38], [39]. Among the genes determining these clustering phenomena, the p21 encoding *Cdkn1a* gene was found in the study by Dahia *et al* [10], whereas the closely related *Cdkn1c*, encoding the cyclin-dependent kinase inhibitor 1C (p57, Kip2), was found differentially expressed in a study by Lopez-Jimenez *et al* [39]. In addition, in a work by Merlo *et al* [40] where paraganglioma tissue was compared with normal paraganglia, *Cdkn1a* was present among a total of 1296 differentially expressed genes. Together, these data support a role of p21 in Sdh-related tumorigenesis in humans.

The fact that a third hit event does not take place in our SDHD-ESR mouse model could be attributable to many circumstances. It could be that the shorter life-span resulting from administration of the minimal effective dose of tamoxifen, i. e., the minimal amount tested to cause reliable deletion of the *SdhD* gene, does not allow subsequent tumorigenic events to occur. Nevertheless, a tissue-specific mutant with a longer life-span than that of the SDHD-ESR model does not show any tendency to form tumors [27]. Therefore, other genetic factors, such as gene redundancy, different chromosomal arrangement, or different susceptibility to the lack of one *SdhD* allele (i.e. haploinsufficiency), could account for these differences between humans and mice.

An alternative role for p21^WAF1/Cip1^ as an onco-protein in Sdh-deficient tissues could also be discussed. Although the well described role of p21^WAF1/Cip1^ as a tumor suppressor opposes a mechanism in which its induction is associated with tumorigenesis [36], [37], it has been demonstrated that under certain conditions, p21^WAF1/Cip1^ can promote cellular proliferation and oncogenicity [36], [41]. Indeed, its overexpression or cytoplasmic localization correlates with poor prognosis in certain malignant tumors ([37] and references therein).

The transcriptional activation of the *Cdkn1a* gene seems to be independent of p53 at least in the SDHD-ESR derived MEFs and BMK cells, as these cells were immortalized by stable expression of a p53-dominant negative protein (see *methods* section) or SV40 large T antigen, which eventually also inactivates p53. Although we do not rule out that in mouse tissues p53 plays a role in *SdhD*-mutation-induced p21^WAF1/Cip1^ induction, such p53-independent expression of p21^WAF1/Cip1^ has been reported in other situations [42]. It is also worth noting a study by Young *et al*., in which acute inactivation of pVHL –the ubiquitin ligase part of the proteasome that downregulates Hif1α under normoxic conditions- caused a senescent-like phenotype in MEFs, with overexpression of p27, another cyclin-kinase inhibitor. This phenotype is independent of p53 and Hif1α, even though Hif1α and Glut1 proteins were accumulated in these cells [43].

Complete loss of *SdhD* induces overexpression of the glucose transporter, Glut1, in our cultured cells, which indicates a metabolic switch towards glycolysis. This change in gene expression seems to be mediated by a “pseudo-hypoxic” response in which Hif1α plays a central regulatory role [11], [12], [13]. However, it is conceivable that, even though the up-regulation of Glut1 may be caused in the first instance by activated Hif1α, the complete loss of mitochondrial function will eventually force the cells to undergo a glycolytic switch with gene expression changes independent of Hif1α. Indeed, a general and rapid Hif-mediated “pseudo-hypoxic” response cannot be addressed from the SDHD-ESR model, as some *bona fide* Hif-target genes are not affected in a general and consistent manner (Table 1). Our cell culture experiments also showed that Hif1α stabilization is transient, which possibly makes difficult to detect this protein in SDHD-ESR tissues after tamoxifen administration. In this regard, it has been demonstrated that reactivation of PHDs takes place upon sustained hypoxia [44]. Although the same could happen in response to *SdhD* deficiency, a marginal contribution of Hif1α to the observed phenotypes cannot be ruled out. Indeed, the microarray analysis of SDHD-ESR tissues did not show a general gene expression profile responsive to hypoxia. Finally, it is noteworthy that we also did not observe any evidence of “pseudo-hypoxia”- driven changes in gene expression in either partially *SdhD*-deficient heterozygous tissues or in derivative cells with the same phenotype. Taken together, our data suggest that a pathogenic role for *SdhD*-mutation-induced Hif1α accumulation cannot be definitively established. Instead, it could play an important role in tumor progression once it has already been formed.

One striking issue regarding tumors caused by mutations in MCII or associated proteins concerns tissue specificity. Although in recent years this has been partially resolved by the fact that Sdh-mutation-related tumors are found in other organs, there is a preferential trend for these types of tumor to arise in paraganglionic system-derived tissues. It has been proposed that an intrinsic ability of these organs to detect oxygen might underlie a predisposition to form tumors [30]. However, other biological characteristics could be equally relevant. Thus, the difference in gene expression changes between the adrenal medulla and kidney found in our study indicates that these tissues respond differently to *SdhD* deletion. In the adrenal medulla, a response pointing towards inhibition of the inflammatory response and immune surveillance is elicited, with changes in the expression of many chemokines, cytokines, and their receptors. The kidney, however, responds in a more “predictable” manner, with many metabolic readjustments promoting cell viability and survival. The physiological relevance of these changes will be explored in future work.

## Conclusions

The identification of p21^WAF1/Cip1^ as one molecule that responds in a general manner to complete *SdhD* deletion, together with its critical role in the cell cycle, senescence, and DNA integrity, paints a new picture of the molecular and cellular responses that take place after MCII dysfunction. The precise mechanism that signals mitochondrial dysfunction to p21^WAF1/Cip1^ activation as well as its cellular effects will be explored in future work. Additionally, a more exhaustive analysis of the large dataset of affected genes generated in this study, and the pathways in which they are involved, will open new avenues towards identifying other processes that could contribute to tumorigenesis and, ideally, will help to identify molecular targets for the treatment of these types of tumors.
